# oREV: An item response theory-based open receptive vocabulary task for 3- to 8-year-old children

**DOI:** 10.3758/s13428-023-02169-3

**Published:** 2023-07-05

**Authors:** Manuel Bohn, Julia Prein, Tobias Koch, R. Maximilian Bee, Büsra Delikaya, Daniel Haun, Natalia Gagarina

**Affiliations:** 1 Department of Comparative Cultural Psychology, Max Planck Institute for Evolutionary Anthropology, Deutscher Platz 6, 04103 Leipzig, Germany; 2 Institut für Psychologie, Leuphana Universität Lüneburg, Lüneburg, Germany; 3 Institut für Psychologie, Friedrich-Schiller-Universität Jena, Jena, Germany; 4 Leibniz-Zentrum Allgemeine Sprachwissenschaft, Berlin, Germany

**Keywords:** Language development, Vocabulary, Individual differences, Item response models

## Abstract

Individual differences in early language abilities are an important predictor of later life outcomes. High-quality, easy-access measures of language abilities are rare, especially in the preschool and primary school years. The present study describes the construction of a new receptive vocabulary task for children between 3 and 8 years of age. The task was implemented as a browser-based web application, allowing for both in-person and remote data collection via the internet. Based on data from *N* = 581 German-speaking children, we estimated the psychometric properties of each item in a larger initial item pool via item response modeling. We then applied an automated item selection procedure to select an optimal subset of items based on item difficulty and discrimination. The so-constructed task has 22 items and shows excellent psychometric properties with respect to reliability, stability, and convergent and discriminant validity. The construction, implementation, and item selection process described here makes it easy to extend the task or adapt it to different languages. All materials and code are freely accessible to interested researchers. The task can be used via the following website: https://ccp-odc.eva.mpg.de/orev-demo.

## Introduction

Individual differences in language abilities are early-emerging, stable across development, and predictive of a wide range of psychological outcome variables including cognitive abilities, academic achievement, and mental health (Bornstein et al., 2018; Marchman & Fernald, 2008; Morgan et al., 2015; Schoon et al., 2010; Walker et al., 1994). From a methodological perspective, high-quality, easy-access measures of language abilities are therefore central to both basic and applied research on individual differences in language abilities. Developing such measures is very time- and resource-intensive, and as a consequence, few exist. In this paper, we describe the construction of a new receptive vocabulary task for German-speaking children. Its theory-driven item generation process makes it linguistically credible. Its psychometric grounding in item response theory (IRT) equips it with the advantages and properties of IRT models (Embretson & Reise, 2013). Its web-based design and implementation make the measure easy to adapt and administer in different settings (in-person or remote) and thereby facilitates the scaling of data collection.

Language has many domains and aspects that can be focused on when assessing individual differences across children. One particular productive approach has been the study of children’s vocabulary skills, that is, their knowledge of word–object mappings. This skill can be most effectively assessed, for example, by asking children to name an object (expressive vocabulary) or pick out an object that matches a word they just heard (receptive vocabulary). Children with larger vocabularies are taken to have advanced language skills more broadly. This assumption seems to be justified in light of strong correlations between vocabulary size and other language measures such as grammatical (Hoff et al., 2018; e.g., Moyle et al., 2007) or narrative skills (Bohnacker et al., 2022; Fiani et al., 2022; Lindgren & Bohnacker, 2022; Tsimpli et al., 2016). Vocabulary skills have also been used as an indicator of developmental language disorders more broadly (Spaulding et al., 2013). Finally, many of the predictive relations found for early language skills mentioned above are based on vocabulary measures (Bleses et al., 2016; Golinkoff et al., 2019; Pace et al., 2017, 2019). This set of findings underlines the importance of high-quality vocabulary measures.

A range of measures exists to assess vocabulary skills in children. For very young children (up to 3 years), a frequently used measure is the MacArthur–Bates Communicative Development Inventories (CDIs) (Fenson et al., 2007). Parents are provided with a list of words and are asked to check those the child understands and/or produces. The CDI exists in different forms (e.g., Makransky et al., 2016; Mayor & Mani, 2019), including an online version (de Mayo et al., 2021), and has been adapted to many different languages (see Frank et al., 2021). Due to concentrated collaborative efforts, data from thousands of children learning dozens of languages have been pooled in centralized repositories (Frank et al., 2017; Jørgensen et al., 2010). As such, the CDI provides a positive example of a high-quality, easy-access measure that is used heavily in both basic and applied research.

However, the CDI is best suited for children in the first two years of life. From 2 years onward, children are usually tested directly. Receptive vocabulary assessment is often part of standardized tests of cognitive abilities (e.g., Bayley, 2006; Gershon et al., 2013; Wechsler & Kodama, 1949). In addition, a range of dedicated measures exist for English (e.g., Dunn et al., 1997; Golinkoff et al., 2017), German (Glück & Glück, 2011; Kauschke & Siegmüller, 2002; Kiese-Himmel, 2005; Lenhard et al., 2015) and other languages.

Yet, from a researcher’s perspective, these existing measures are often problematic for several reasons. Because they are standardized and normed instruments, using them comes with substantial licensing costs. For the same reasons, the corresponding materials are not openly available, which makes it difficult to expand or adapt them to different languages. Most measures also rely on in-person, paper-and-pencil testing, which makes large-scale data collection inefficient. Whenever more portable, computerized versions exist, they come with additional costs. As a consequence, nothing comparable to the collaborative research infrastructure built around the CDI exists for vocabulary measures for older children.

The development of so-called Cross-linguistic Lexical Tasks [CLTs; Haman et al. (2015)] constitutes a promising framework that might help to overcome these issues. CLTs are picture-choice and picture-naming tasks aimed at assessing receptive and expressive knowledge of nouns and verbs in children up to 5 years of age. In a collaborative effort involving more than 25 institutions, versions for dozens of different languages have been developed following the same guiding principles (Armon-Lotem et al., 2015; Haman et al., 2015, 2017). In addition to cross-linguistic studies with monolingual children, this procedure makes CLTs ideally suited to assess multilingual preschool children. The tasks and the materials are not commercially licensed and can thus be freely used for research purposes.

Despite these many positive characteristics, CLTs are limited in two important ways. First, they were designed for children between 3 and 5 years of age and consequently show ceiling effects for older children in this age range (Haman et al., 2017). This greatly limits their usefulness in research across the preschool years. Second, and perhaps more important, CLTs have been developed following clear linguistic guidelines—but *without* a strict psychometric framework^1^. As a consequence, it is unclear how the different items relate to the underlying construct (e.g., vocabulary skills). We do not know which items discriminate between varying ability levels and are therefore particularly diagnostic e.g., at different ages. Items could also be biased and show differential measurement properties in relevant subgroups (e.g., girls and boys). In addition, some items might be simply redundant in that they measure the underlying construct in the same way. Such characteristics could make the task unnecessarily long. Modern psychometric approaches like IRT (Kubinger, 2006; Lord, 2012) allow researchers to adequately model the probabilistic relationship between the items of a test and the underlying latent trait. In addition, it can be empirically tested how well the individual items are suited to capture a latent dimension and what psychometric properties are associated with the specific test. This focus allows for evaluating the quality and usefulness of each item and thereby provides a solid psychometric basis for constructing efficient and high-quality tasks. In combination with a computerized implementation, IRT allows for adaptive testing during which participants are selectively presented with highly informative items given their (constantly updated) estimated level of ability. However, IRT-based task construction requires a higher initial investment: it takes a large item pool and large sample sizes to estimate the item parameters that guide the selection of the best items.

## The current study

Our goal was to develop a theoretically grounded, high-quality, easy-access measure of receptive vocabulary skills for German-speaking children between 3 and 8 years of age. For this purpose, we built on the existing CLT but substantially expanded the item pool. We implemented the task as a browser-based web application, which made it highly portable and allowed us to test a large sample of children online. Next, we used IRT to estimate measurement characteristics of each item in the pool. We then developed an algorithm that used these characteristics to automatically select a smaller subset of items for the final task. The implementation infrastructure and construction process we describe here make the task easy to share with interested researchers and practitioners and also provide clear guidance on how to further adapt it to different languages.

## Access to data and materials

The data sets generated during the current study and the analysis code are available in the following repository: https://github.com/ccp-eva/orev. The task (after item selection) can be accessed via the following link: https://ccp-odc.eva.mpg.de/orev-demo. Finally, the source code, pictures, and sound files used in the task can be accessed via the following repository: https://github.com/ccp-eva/orev-demo.

## Item-pool generation

The initial item pool consisted of 32 items taken with permission from the German CLT (Haman et al., 2015, 2017) and 20 new items. The addition of new items was necessary due to ceiling effects for monolingual 5-year-olds in the previous version. New items were generated in line with the construction of the original CLT in a stepwise process. Each item consists of a target word and three distractors. To select target words, we first compiled a list of age-of-acquisition ratings for 3928 German words from various sources (Birchenough et al., 2017; Łuniewska et al., 2019; Schröder et al., 2012). From this list, we selected 20 words based on the following criteria: words should refer to concepts that could easily and unambiguously be depicted in a drawing, age-of-acquisition ratings should be spread equally between 6 and 10 years of age. We also computed (semantic) complexity indices for each word (see Haman et al., 2017). This metric, however, did not reflect a dimension that was relevant for item selection.

The so-selected 20 words served as additional target words in the item pool (total of 52 items). For each target word, we selected three distractors. The first distractor was unrelated to the target word but was chosen to have a comparable rated age of acquisition. The second distractor was semantically related to the target word (e.g., ruin–fortress; elk–mammoth). The third distractor was phonetically similar to the target. For example, the initial part was substituted, while the rest of the word was kept similar (e.g., Gazelle [eng.: gazelle]–Libelle [eng.: dragonfly]). The complete list of targets and distractors can be found in the associated online repository. Finally, an artist (same as for the original CLT items) drew pictures representing all target and distractor words. This procedure ensured that the original CLT and the newly generated items formed a homogeneous item pool.

## Task design and implementation

The task was programmed in JavaScript, CSS, and HTML and presented as a website that could be opened in any modern web browser. In addition to participants’ responses, we recorded webcam videos^2^. Both files were sent to a local server after the study was finished. The task started with several instruction pages that explained to parents the task and how they should assist their child if needed.

**Fig. 1:**
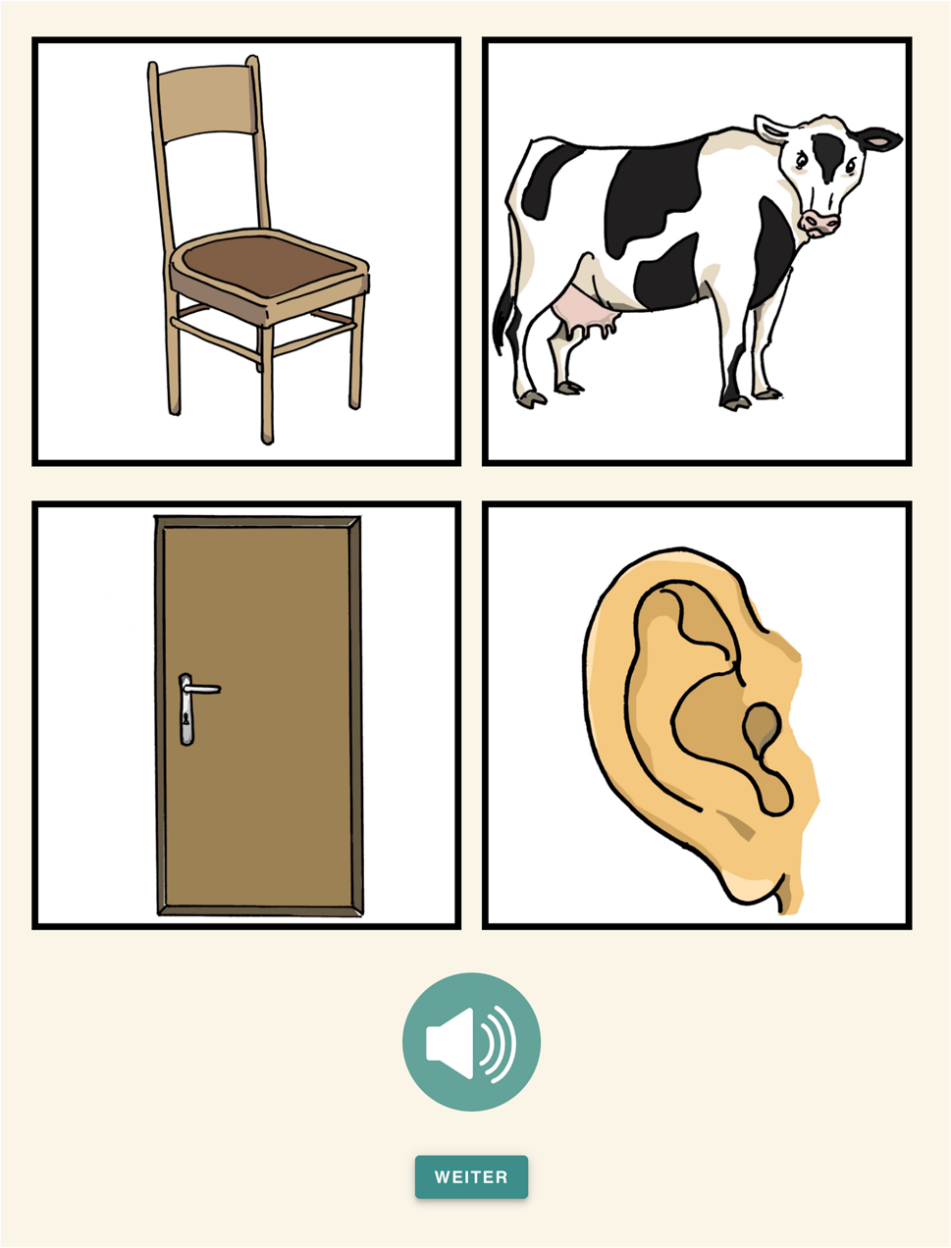
Screenshot of the task. On each trial, participants heard a word and were asked to pick out the corresponding picture. Verbal prompts could be replayed by pressing the loudspeaker button

On each trial (see Fig. 1), participants saw four pictures and heard a verbal prompt (pre-recorded by a native German speaker) asking them to select one of the pictures (prompt: “Zeige mir [target word]”; eng.: “Show me [target word]”). The verbal prompt was automatically played at the beginning of each trial. The prompt could also be replayed by clicking on a loudspeaker button if needed. Pictures could only be selected once the verbal prompt finished playing. Selected pictures were marked via a blue frame. Participants moved on to the next trial by clicking on a button at the bottom of the screen. If children could not select the pictures themselves (via mouse click or tapping on the touch screen), they were instructed to point to the screen and parents should select the pointed-to picture.

The positioning of the target was counterbalanced across four positions (upper/lower and left/right corners) according to three rules: (1) the target picture appeared equally often in each position; (2) the target picture could not appear in the same position in more than three consecutive trials; (3) the target picture appeared in each position at least once across seven subsequent trials. Distractors were distributed across the remaining three positions so that each distractor type (i.e., unrelated, phonological, semantic) appeared equally often in each position across trials. We generated two versions of the task with different item orders. Each order was created so that trial number and age-of-acquisition ratings were correlated with *r* = .85. This would make later trials more difficult, but not perfectly so.

## Item selection

The goal of the item selection process was to find a subset of high-quality items necessary to measure vocabulary skills on an individual level. As a first step, we collected data for the full 52-item task from more than 500 children in the target age range. Next, we fit a Rasch (one-parameter logistic [1PL]) and a 2PL IRT model to the data to estimate parameters of interest for each item which we used during the item selection process. We used a simulated annealing process (Kirkpatrick et al., 1983) to simultaneously determine the size of the reduced task and to select the best items. Our goal was to construct a reduced task that (a) included items of varying difficulty and (b) fit the Rasch model so that an individual’s test score (number of solved items) was a sufficient statistic and the task was easy to use. After selecting items and constructing the new task, we conducted visual model checks and investigated differential item functioning (DIF) when the data were split either by sex or by trial order. Data collection was pre-registered at https://osf.io/qzstk. The pre-registered sample size was based on recommendations found in the literature (Morizot et al., 2007). However, these authors emphasize that the necessary sample size depends very much on the complexity of the model and that recommendations should be treated with caution.

### Participants

Participants were recruited via a database of children living in Leipzig, Germany, whose parents volunteered to participate in studies on child development and who additionally indicated interest in participating in online studies. Leipzig is an industrialized, urban Central European city with approximately 600,000 inhabitants. The city-wide median individual monthly net income in 2021 was ~ €1600. Children mostly live in nuclear two-generational families. Socioeconomic status was not formally recorded, although the majority of families come from mid to high socioeconomic backgrounds with high levels of parental education. Furthermore, it is very likely that selective responding skewed the sample towards highly motivated and interested families. Parents received an email with a short study description and a personalized link. After one week, parents received a reminder if they had not already taken part in the study. Response rate to invitations was ~50%. The final sample included a total of 581 children (*n* = 307 girls) with a mean age of 5.63 years (range: 3.01–7.99). Participants were randomly assigned to one of the two item orders. Data were collected between February and May 2022.

### Descriptive results

**Fig. 2:**
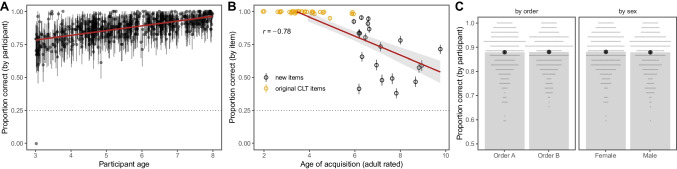
Descriptive results of the task. **A** Proportion of correct responses (with 95% CI) for each participant by age. **B** Proportion of correct responses (with 95% CI) for each item by rated age of acquisition of the target word. **C** Proportion of correct responses (with 95% CI) by trial order (left) and sex (right).

On a participant level, performance in the full task (52 items) steadily increased with age (Fig. 2A). On an item level, performance was above chance (25%) for all items. Furthermore, the average proportion of correct responses was negatively correlated with age-of-acquisition ratings (Fig. 2B) and positively correlated (*r* = 0.31; 95% CI = 0.02–0.55) with the normalized frequency of the word in children’s books reported in the childLex corpus (Schröder et al., 2015). Figure 2B also shows the ceiling effect for the original CLT items found in Haman et al. (2017). These descriptive results replicate well-known results in the literature and emphasize the added value of the newly developed items. Figure 2C shows that there were—on average—no differences between participants who received order A and order B nor between female and male participants. This result suggests that these grouping variables are suitable to investigate differential item functioning (see below).

### Item response modeling

IRT models were implemented in a Bayesian framework in R using the brms package (Bürkner, 2017, 2019). Given the binary outcome of the data, we used logistic mixed-effects models to predict the probability of a correct answer based on the participant’s latent ability and item characteristics. We fit two models: a Rasch (1PL) model and a Birnbaum (2PL) model. The main difference between these two models lies in their assumption about item discrimination, that is, how the probability of solving an item changes with ability levels. While the Rasch model assumes that the rate of change (i.e., the slope of the logistic curve) is the same for all items, the 2PL model estimates a separate discrimination parameter for each item. Only when items have similar discrimination parameters is the sum score a sufficient statistic. Given the structure of the task (selecting one out of four pictures), both models had a fixed guessing rate of 0.25^3^. All models converged properly according to visual inspection (e.g., traceplots) and convergence diagnostic measures (e.g., Rhat close to 1, Vehtari et al., 2021). For details about prior and MCMC settings, please see the analysis script in the associated online repository.

For each item, we computed the following parameters to be used during the item selection process: Difficulty (parameterized as easiness, i.e., the additive inverse of difficulty) according to the Rasch model, In- and Outfit based on the Rasch model and item discrimination according to the 2PL model. Difficulty estimates represent the level of ability (point on the latent dimension) for which the probability of solving an item is .5. In- and Outfit are calculated based on the deviation of a person’s response to an item from the response predicted by the model according to their level of ability and item difficulty. As such, they reflect how well the Rasch model captured the responses to a particular item. As noted above, item discrimination parameters in the 2PL model influence the rate at which the probability of solving an item changes given ability levels. In the next section, we describe how we used these parameters to select items.

### Automated item selection

The item selection process focused on selecting a smaller subset of items that fit the Rasch model and allowed for precise measurement at different levels of receptive vocabulary ability. Only when items fit the Rasch model is the number of solved items a sufficient statistic for an individual’s ability. Being able to use the sum score—instead of estimating person parameters via a model—makes the task very easy to use. For this purpose, we defined an objective function that captured three important characteristics that the items of any subset should have. First, items should be equally spaced across the latent ability space. This characteristic ensures that the task is suited for different ability levels and thus for a broader range of ages. We quantified the spread of any given subset as the standard deviation of the distance (in easiness estimates) between adjacent items. Lower values indicate smaller distances and thus an overall more equal spacing. Second (and third), items should have In- and Outfit values close to 1. In- and outfit values that deviate from 1 indicate over- or under-fitting and suggest that the respective item is not in line with the Rasch model; conversely, the smaller the value, the better. Finally, items should have similar discrimination parameters according to the 2PL model. The Rasch model assumes that all items have the same discrimination, and thus selecting items with similar discrimination parameters in the 2PL model ensures a better fit of the Rasch model. We quantified this aspect as the variance of discrimination parameters of a given subset of items. Lower variances indicate more similar discrimination parameters and a better fit of the Rasch model.

Next, we multiplied/divided these values by constants to put them on a similar numeric scale and to emphasize some aspects over others. We put special emphasis on In- and Outfit values (to select items that conform to the Rasch model) as well as on the equal spacing of items across the latent dimension (to select items suitable for different ages). Details and data simulations can be found in the analysis script in the associated repository. Finally, we defined the objective function as the sum of the scaled parameters.

We used simulated annealing (Kirkpatrick et al., 1983) to find the optimal items for any given subset size. This process randomly explores the large space of possible subsets, starting from a randomly selected initial subset. Then, it proposes small random changes by exchanging some items in the subset under consideration with others outside it. If such a change increases the value of the objective function, the proposal is accepted, and the improved subset is taken as the new starting point for subsequent proposals. However, to avoid the process getting trapped in local optima, proposals that decrease the value of the objective function may also be accepted, but probabilistically. The probability that a proposal decreasing the objective function is accepted depends upon a parameter called “temperature”, which is gradually reduced from a high initial value to a lower value over the course of the simulation. During the “hot” early phase, the process explores the space relatively freely, accepting decreasing proposals often enough to allow it to move between local optima separated by less well-performing subsets, facilitating the discovery of global optima. In the later “cool” phases, the process slowly converges to a strict “hill-climbing” search that accepts only increasing proposals, resulting in careful fine-tuning of the best subset discovered in the hot phase.

We applied simulated annealing to subsets ranging from 10 to 40 items. For each (optimal) subset, we fit a Rasch model and a 2PL model and compared them using Bayesian approximate leave-one-out cross-validation (Vehtari et al., 2017) based on differences in expected log posterior density (ELPD) estimates and the associated standard error (SE). This method of comparison balances between fit to the data and out-of-sample predictive accuracy and thereby adjusts for model complexity. Therefore, it favors neither underfitting (because predictions would be too rigid) nor overfitting (because predictions would only match the sample). We considered models to be equivalent up to a point when the ELPD in favor of the 2PL model exceeded two times the standard error of the difference. This rule of thumb is based on suggestions in the literature but is by no means a hard-and-fast cutoff (Sivula et al., 2020). In addition, we computed the correlation between performance based on the subset and the full task.

**Fig. 3:**
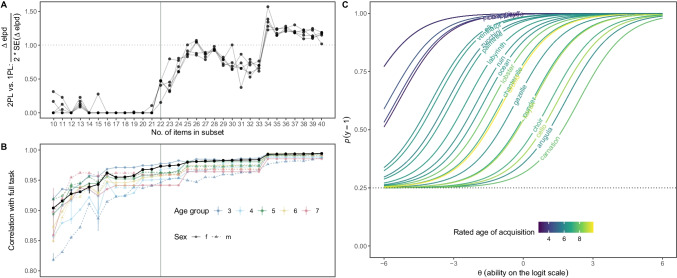
Item selection process. **A** Model comparison ratio comparing the fit of a Rasch model to the fit of a 2PL model for different sizes of the subtest. The *y*-axis label shows how the ratio is computed. Values of 0 indicate a better fit of the Rasch model compared to the 2PL model. The dashed line marks a ratio of 1, which we assumed to be the point when the 2PL model clearly provided a better fit. Points and lines show the results from five independent runs of the model comparison procedure. **B** Correlation between reduced and full task (52 items). Points show mean correlation based on five iterations. Vertical lines show the range of correlations in cases when they differed between iterations. Black lines and points show correlations for the full sample, and colored points and lines show correlations by age group and sex. **C** Item characteristic curves for the 22 colored by their rated age of acquisition.

Our goal was to find the optimal size and items for the subtest. Figure 3A visualizes the model comparison ratio and shows that the fit of the Rasch model compared to the 2PL model decreases substantially for subsets with more than 24 items. Figure 3B visualizes the correlation between the subtest and the full task, both across all individuals and separately by age and sex. Even though correlations were generally high, they reached a plateau at around 20 items. Based on these results, we chose a size of 22 items for the subtest. For 22 items, the ELPD difference (in favor of the 2PL model) ranged from −0.86 (SE of difference = 2.81) to −2.77 (SE of difference = 2.92). This suggests that the two models were more or less equivalent at that point and that freely estimating the discrimination parameters did not improve the model fit substantially. Thus, the Rasch model provides a good absolute fit for the 22 selected items. Even though a smaller subtest would have been justifiable (e.g., 20 or even 18), we decided to include more items to allow for more precise individual-level measurements. We acknowledge, however, that this decision is to some extent arbitrary.

When running the simulated annealing procedure for 22 items 100 times, it always returned the same item selection. We, therefore, chose this subset of items for the reduced task. The so-constructed task correlated highly with the full task, both across participants and when the data were split by age group and sex (see Fig. 3B)^4^. The selection procedure via the simulated annealing algorithm ensured that the items were equally spread across the latent dimension (see Fig. 3C for item characteristic curves).

### Differential item functioning

**Fig. 4:**
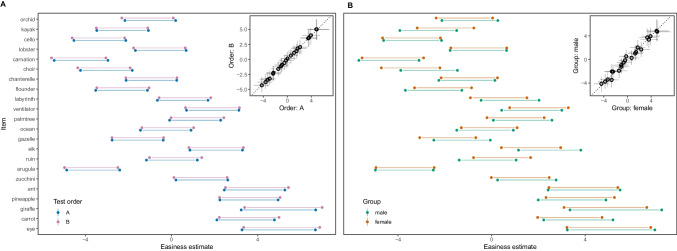
Easiness estimates for all items (ordered by rated age of acquisition) of the newly constructed subtest separate for each test order (**A**) and sex (**B**). Dots connected by lines show 95% CrI, color denotes the subgroup. Insets show correlations between the parameters for each subgroup based on the mode of the posterior distribution for each item.

Next, we fit two additional Rasch models in which we estimated separate difficulty parameters for two subgroups: one for sex (male and female) and one for the order in which items were presented (order A or order B). This allowed us to assess the absolute fit of the Rasch model and to assess differential item functioning (DIF, see Bürkner, 2019). DIF refers to situations where items show differential characteristics for subgroups that otherwise have the same overall score (Holland & Wainer, 2012). If the Rasch model fits the data well and no item shows DIF, the estimates based on the two subgroups should be very similar. Figure 4 shows that this was clearly the case for all items, regardless of whether the data were split by test order or sex. As a consequence, we can say that the newly constructed test was very well described by the Rasch model so that the number of solved items represents a sufficient statistic for an individual’s vocabulary skills.

## Psychometric properties of newly constructed task

### Reliability

We computed two reliability indices for the newly constructed oREV task: KR-20 (Kuder & Richardson, 1937) and Andrich reliability (Andrich, 1982). Both indices indicated good reliability for the subtest (KR-20 = 0.76; Andrich = 0.74) that were comparable to the full test (KR-20 = 0.78; Andrich = 0.77).

### Stability

We were able to re-test 184 children (88 girls; mean age first testing = 4.96; range = 3.03–6.99) approximately one year (mean number of days between testing = 341; range = 302–369) after the initial data collection with the newly constructed task. Parents received personalized links and children were tested online. As expected, overall performance in the sample increased from 73% to 80% correct, showing developmental gains in receptive vocabulary with age. Nevertheless, individual differences were stable: performance was strongly correlated between the two time points (*r* = 0.67; 95% CI = 0.58–0.74).

### Convergent and discriminant validity

**Fig. 5:**
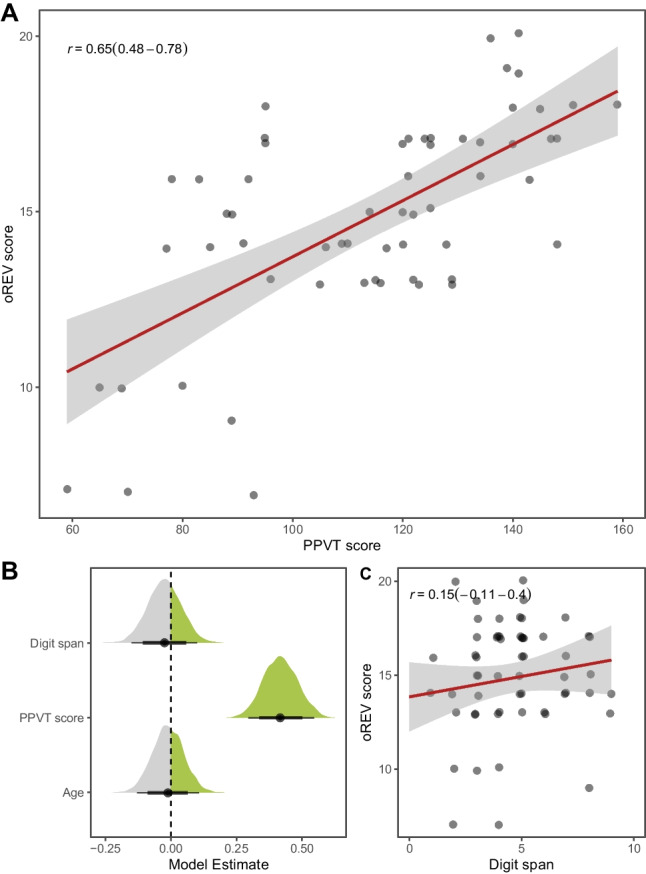
Convergent and discriminant validity. **A** Pearson correlation (with 95% CI) between PPVT and oREV scores. **B** Posterior model estimates for digit-span, PPVT scores, and age in a model predicting oREV scores. Points show posterior means with 95% CrI. **C** Pearson correlation (with 95% CI) between digit-span and oREV scores. In (**A**) and (**C**): Gray points show individual data points with minimal horizontal and vertical noise added to avoid overplotting. Red lines show regression lines (with 95% CI) based on a simple linear model.

Finally, we assessed convergent and discriminant validity of our task. We used the Peabody Picture Vocabulary Test (PPVT; Dunn & Dunn, 2007; Lenhard et al., n.d.) as a convergent measure of receptive vocabulary and the digit-span task from the K-ABC (Lichtenberger et al., 2009) as a discriminant measure of working memory. These two tasks were unavailable as online versions, and we, therefore, turned to in-person data collection. We tested 59 children in kindergartens around Leipzig, Germany. We chose a relatively narrow age range (mean = 5.54; range = 4.97–6.02) to avoid strong correlations between tasks due to general developmental gains. Data were collected between January and May 2023. The oREV scores were highly correlated with PPVT scores (*r* = 0.65; 95% CI = 0.48–0.78), but not with digit-span scores (*r* = 0.15; 95% CI = −0.11 to 0.40). Conversely, when we predicted the number of correctly solved items in the oREV by PPVT scores, digit-span scores, and age in a binomial model, PPVT scores had by far the strongest influence (Fig. 5). Taken together, these results demonstrate the convergent and discriminant validity of the oREV task.

## Discussion

Individual differences in language abilities in childhood are an important predictor of later life outcomes. Yet, high-quality, easy-access measures are rare, especially for pre- and primary school-age children. Here we reported the construction of a new receptive vocabulary task for German-speaking children between 3 and 8 years of age. Building on earlier work (Haman et al., 2017), we first generated a larger initial pool with 52 items. Next, we implemented the picture-selection task as a web application and collected data from over 500 children online. We used IRT models and an automated item selection algorithms to select a set of high-quality items that fit the Rasch model. The so-constructed task has 22 items of varying difficulty, correlates with the full task at a rate of .97, and shows good reliability and stability. Furthermore, high correlation with a theoretically related task and low correlation with an unrelated task illustrate its convergent and discriminant validity. Its browser-based implementation makes the task highly portable and facilitates large-scale data collection. The construction and item selection process we described here makes it easy to add additional items or adapt the task to different languages while retaining a high psychometric quality of the end product. The task is freely accessible to all interested researchers.

The task fills an important gap in the methods repertoire of developmental researchers studying language development in early childhood. Existing measures show ceiling effects, come with high licensing costs, and/or are not available in an electronic format. Our task captures variation between children up until 8 years of age, is free to use, and can be run on any modern web browser. However, the newly constructed task with 22 items is still relatively easy, that is, most 7-year-old children will solve the majority of items (89% correct responses in the present sample). As a consequence, it does not distinguish well between children with very strong vocabulary skills. Future extensions of the task could thus focus on adding more difficult items. Figure 2B (see also Brysbaert & Biemiller, 2017) shows that target word age-of-acquisition ratings are a fairly good predictor of item difficulty and could be used as a basis to generate new items. Extensions should focus on target words with rated age of acquisition above 10. Further extensions could target other parts of speech, such as verbs and adjectives.

The sample we tested to construct the test was not representative (in terms of socioeconomic status) and likely skewed to children with higher language proficiency than average. As a consequence, the task and the data set should not be used for diagnostic purposes but only as a research tool to capture variability in a population of interest. Nevertheless, the good psychometric properties of the task make it an ideal candidate for future norming studies with representative samples.

The automated item selection process we implemented critically leveraged the strengths of IRT modeling. For each item in the pool, we estimated its difficulty and fit to the Rasch model. The objective function we optimized via the simulated annealing process was defined so that it would yield a subset in which items would (a) be equally spread out across the latent ability so that the task measured equally well at different skill levels and (b) fit the Rasch model so that the sum score is a sufficient statistic for the ability parameter.

This procedure presents a principled way of constructing a task with good psychometric properties, which can be easily applied to any new set of items or versions of the task in different languages. However, this approach does not make the careful, principle-based construction of the initial item pool superfluous; it only selects the best of the available items. Linguistic and psychometric considerations thus need to go hand in hand during task construction. For example, while nouns are more similar across languages, verbs are more language-specific and might have different representations or even be absent as a single word. For example, the German verb “wandern” (eng: “hiking”) can only be expressed by an analytical construction in the majority of Slavic languages. Furthermore, bilingual and monolingual lexicons might vary, and background factors such as length of exposure, the onset of second language acquisition, or birth order should be considered. Finally, language-specific morphosyntactic properties of grammar, such as perfective or imperfective aspect in verbs, should be taken into account.

A major advantage of the task presented here is its portability. Its implementation as a web application makes it easy to administer both in person and online and also reduces the likelihood of experimenter error. In fact, we were able to collect data from more than 500 children online in just two months. It is also easy to add new items or to adapt the existing task to a new language. Of course, extensions and new adaptations require a renewed item evaluation and selection process. Nevertheless, the infrastructure and materials developed here provide a good starting point for such an endeavor. The computerized implementation of the task also allows for adaptive testing. Instead of all participants completing the same set of items, each participant could be presented with—potentially fewer—maximally informative items given their (continuously updated) estimated skill level. However, this would require a more elaborate back-end—capable of doing online parameter estimation—compared to the current version of the task.

## Conclusion

We have described the construction of a new receptive vocabulary task for German-speaking children between 3 and 8 years of age. The task has good psychometric properties and shows convergent and discriminant validity. We believe it is an important research instrument to measure individual differences in receptive vocabulary skills. The task, and the materials it is constructed from, are openly available. As such, it closes a prominent gap in the toolkit of developmental researchers.

## Data Availability

All data sets generated during the current study and the corresponding analysis code are available in the following repository: https://github.com/ccp-eva/orev.

## Footnotes

1 The same applies to most other vocabulary measures used in developmental research.

2 Due to access rights issues, webcam recording was not possible when participants used iOS devices.

3 In the Rasch model, the number of solved items is still a sufficient statistic for an individual’s ability when there is a fixed guessing rate (see Jiao, 2022).

4 The high correlations are not surprising given that 17 of the 22 selected items were newly added items. The ceiling effect for most of the original CLT items meant that most of the variation between individuals was captured via the newly added items. Any test with many of the newly added items would have a high correlation with the full task. For this reason, we did not include the correlation with the full test in the objective function passed on to the simulated annealing algorithm.
